# High Awareness but Low Coverage of a Locally Produced Fortified Complementary Food in Abidjan, Côte d’Ivoire: Findings from a Cross-Sectional Survey

**DOI:** 10.1371/journal.pone.0166295

**Published:** 2016-11-08

**Authors:** Magali Leyvraz, Fabian Rohner, Amoin G. Konan, Lasme J. C. E. Esso, Bradley A. Woodruff, Augusto Norte, Adiko F. Adiko, Bassirou Bonfoh, Grant J. Aaron

**Affiliations:** 1 Global Alliance for Improved Nutrition, Geneva, Switzerland; 2 GroundWork, Fläsch, Switzerland; 3 Centre Suisse de Recherches Scientifiques en Côte d’Ivoire, Abidjan, Côte d’Ivoire; 4 Université Félix Houphouët-Boigny, Abidjan, Côte d’Ivoire; 5 Swiss Tropical and Public Health Institute, University of Basel, Basel, Switzerland; Institut de recherche pour le developpement, FRANCE

## Abstract

Poor complementary feeding practices among infants and young children in Côte d’Ivoire are major contributing factors to the country’s high burden of malnutrition. As part of a broad effort to address this issue, an affordable, nutritious, and locally produced fortified complementary food product was launched in the Côte d’Ivoire in 2011. The objective of the current research was to assess various levels of coverage of the program and to identify coverage barriers. A cross-sectional household survey was conducted among caregivers of children less than 2-years of age living in Abidjan, Côte d’Ivoire. Four measures of coverage were assessed: “message coverage” (i.e., has the caregiver ever heard of the product?), “contact coverage” (i.e., has the caregiver ever fed the child the product?), “partial coverage” (i.e., has the caregiver fed the child the product in the previous month?), and “effective coverage” (i.e., has the caregiver fed the child the product in the previous 7 days?). A total of 1,113 caregivers with children between 0 and 23 months of age were interviewed. Results showed high message coverage (85.0%), moderate contact coverage (37.8%), and poor partial and effective coverages (8.8% and 4.6%, respectively). Product awareness was lower among caregivers from poorer households, but partial and effective coverages were comparable in both poor and non-poor groups. Infant and young child feeding (IYCF) practices were generally poor and did not appear to have improved since previous assessments. In conclusion, the results from the present study indicate that availability on the market and high awareness among the target population is not sufficient to achieve high and effective coverage. With market-based delivery models, significant efforts are needed to improve demand. Moreover, given the high prevalence of malnutrition and poor IYCF practices, additional modes of delivering IYCF interventions and improving IYCF practices should be considered.

## Introduction

The prevalence of malnutrition in infants and young children in Côte d’Ivoire is high. A national survey conducted in 2007 found that 72% of children between 6 and 59 months were anemic, 24% were vitamin A deficient, 15% had iron deficiency, 41% were stunted, 14% were wasted, and 29% were underweight [1]. A more recent national survey conducted in 2011–2012, which assessed the prevalence of anemia, stunting, wasting, and underweight, indicated some progress, but persistently high prevalence rates of malnutrition: 75% of children between 6 and 59 months were anemic, while 30% were stunted, 8% were wasted, and 15% were underweight [2].

It is widely recognized that exclusive breastfeeding provides the optimal diet for children under 6 months [3]. However, after 6 months of age, the nutritional requirements of infants increase for all macro- and micronutrients such that breast milk alone can no longer cover the infants’ needs [4, 5]. Thus, to ensure adequate nutrition and healthy growth outcomes, it is essential that a variety of nutrient-dense foods be introduced into children’s diets to complement breast milk. The main strategies to increase nutrient density in children’s diet are dietary diversification, supplementation, and fortification [6]. The appropriateness and suitability of a particular approach depends on the context and nutritional needs of the target population as discussed elsewhere [6].

Infant and young child feeding (IYCF) practices are poor in Côte d’Ivoire: Only 12% of children are exclusively breastfed until 6 months of age [7] and fewer than 5% of the children between 6 and 23 months are fed according to the World Health Organization’s optimal feeding recommendations [8].

In 2009, the Global Alliance for Improved Nutrition (GAIN) initiated a project in Côte d’Ivoire to introduce a nutritious and affordable fortified complementary food product targeted at poorer children 6–23 months and to raise awareness and improve IYCF practices. The project was entitled “Projet de Promotion de l’Alimentation de Complément Enrichie du Jeune Enfant en Côte d’Ivoire” (PACE) (Project to Promote the Feeding of Fortified Complementary Foods to Young Children in Côte d’Ivoire) [9] and was led by a non-governmental organization, Helen Keller International (HKI), and a local producer, Protein Kissée-La (PKL).

PACE took an existing product (Farinor) that had been developed for all children 6–23 months in Côte d’Ivoire and reformulated it in accordance with international recommendations into a new product (Nutribon), that was targeted to the country’s poorer consumers. In 2014, PKL produced a total of 181 MT of fortified complementary foods and distributed it through pharmacies (50%), supermarkets (34%), and shops (14%) (personal communication from PKL). In addition, mobile salespersons on motorbikes reached areas that were less served by more traditional commercial means.

HKI conducted formative research on caregivers’ knowledge and practices of optimal feeding. The results of this research showed that knowledge was generally low and practices poor, that coverage of instant cereals was largely limited to wealthier populations in urban areas, and that there was an unmet demand for affordable and nutritious complementary food options [10]. These findings were used by HKI to develop a behavior change campaign that started concurrently with the launch of Nutribon in 2011 (and ended in 2013) and that consisted of a general awareness-raising campaign, training of health workers and community leaders, and cooking demonstrations.

The primary objectives of the current research were to determine the coverage of the PACE program among children 6–23 months of age living in Abidjan, identify the major barriers to coverage of the program, and formulate recommendations for future program activities. Younger children (<6 months) were included to assess IYCF practices and whether the product was inappropriately being consumed.

## Materials and Methods

### Study design and setting

A cross-sectional cluster-based household survey was conducted from September to October 2014. The survey was carried out in Abidjan, as this constituted the main market for PKL’s sales, with more than 75% of the products sold there (personal communication from PKL). Moreover, about 20% of the population of Côte d’Ivoire lives in Abidjan [11].

In the first stage of sampling, nine primary sampling units (PSUs), which consisted of the smallest census unit and contained about 200 households, were randomly selected in each of the 10 communes in Abidjan with the probability of selection for each PSU being proportional to the number of households in that PSU. Prior to the second stage of sampling, a census was carried out in each selected PSU to identify eligible children and households and to estimate population size. The household list in each PSU was used in the second stage of sampling, which consisted of a random selection of 13 households with at least one child aged 0–23 months. If more than one eligible child lived in a selected household, only one child was randomly selected, using a Kish table [12].

### Ethical considerations

The survey protocol was approved by the Comité National d’Éthique et de la Recherche (National Ethics Committee of Côte d’Ivoire), clearance number 55/MSLS/CNR-dkn. Written informed consent was sought from the caregiver of each selected child. All participating households were given two bars of soap and an information brochure on adequate IYCF practices to thank them for their participation.

### Survey instrument and indicator definitions

The survey questionnaire was adapted from a questionnaire that was part of a survey previously conducted in Ghana [13] and was pilot tested. An interview with the main caregiver of the selected child was conducted using the questionnaire and the following data was collected on all household members: sex, age, and education; household socio-demographic status; water, sanitation, and hygiene practices; IYCF practices; women's and children's dietary diversity [14]; and coverage of PKL’s products. Mid-upper arm circumference (MUAC) of the caregiver and the child was measured with a MUAC tape.

A multidimensional poverty index (MPI) was constructed according to Alkire and Santos [15], whereby living standards, education, health and nutrition, and household assets were combined to create an index ranging from 0 (indicating no poverty) to 1 (indicating maximum poverty); a household with an MPI of 0.33 or more was classified as poor.

Severe acute malnutrition (SAM) was classified using the MUAC measurement result. Children under 6 months of age with a MUAC below 110 mm, children 6 months of age and above with a MUAC below 115 mm, and adult women with a MUAC below 230 mm were defined as having SAM [16]. Any caregiver or child diagnosed with SAM was referred to the nearest health facility and follow-up was ensured by a survey nurse hired for this purpose. Household hunger was assessed using the Household Hunger Scale (HHS) [17]. The HHS is an indicator of household hunger that is focused on the food quantity dimension of food access. The questions ask whether or not a specific condition associated with the experience of food insecurity ever occurred during the previous 4 weeks (30 days).

IYCF practices were assessed using the Infant and Child Feeding Index (ICFI) according to Guevarra et al. [18]. This index combines age-specific scores for breastfeeding, child’s dietary diversity and meal frequency patterns into an age-appropriate child feeding practices score which ranges between 0 and 6. Children with an ICFI score of 6 were considered as having optimal IYCF practices; children with an ICFI of less than 6 were considered having poor IYCF practices.

Improved drinking water source was defined as piped water or water from a borehole, protected or semi-protected dug well, protected spring, or rainwater collection. Safe drinking water is defined as using water from an improved source or, in the case of use of unimproved water sources, using adequate home treatment methods to make water safer to drink (i.e., by boiling, adding bleach/chlorine, using a water filter, or using solar disinfection). Adequate sanitation facilities were defined as a flush toilet that pours to a piped sewer, a septic tank or pit latrine, a ventilated improved pit latrine, a pit latrine with slab, or a composting latrine, and not sharing the facilities with non-household members.

The definitions of coverage were based on the model of Tanahashi [19]. This model identifies sequential levels through which coverage is achieved and each level relates to an important step on the pathway to the provision of the intervention [20]. In this context, we defined the following four different coverage levels: “message coverage” (i.e., the caregiver has ever heard of Farinor or Nutribon), “contact coverage” (i.e., the caregiver has ever given the participating child Farinor or Nutribon), “partial coverage” (i.e., the caregiver has given the participating child Farinor or Nutribon at least once in the past month), and “effective coverage” (i.e., the caregiver has fed the participating child Farinor or Nutribon at least once in the past 7 days). In addition, two summary measures were calculated: “met need” (i.e., the proportion of children considered at-risk of poverty or poor IYCF practices that have the coverage measures described above) and “coverage ratio” (i.e., the ratio of a measure of coverage in children considered at-risk to that measure of coverage in children considered not at-risk) [13].

### Data entry and analysis

Double data entry was conducted by dedicated data entry clerks using Epidata (Version 3.1) [21]. Data were examined for missing values and data distributions for normality in the whole sample. Statistical weights applied during all analyses accounted for: the differences between estimated PSU populations used in first-stage sampling and the actual PSU population counted by the survey teams, the different likelihood of selection of a child resulting from the different number of eligible children in households, and the different sampling fraction in the 10 communes. Cluster and stratified sampling were accounted for when calculating measures of precision.

The statistical significance of associations between categorical variables, including the measures of coverage of PKL products, was assessed using adjusted chi-square p values. Adjusted student’s t-test and one-way analysis of variance (ANOVA) were used for assessing the statistical significance of any differences between categorical and continuous variables. P values below 0.05 were considered statistically significant. Multivariate regression analysis was employed to determine independent associations between variables and coverage. Percent met need and coverage ratios were calculated using a blocked weighted bootstrap estimation technique [22] and a total of 400 bootstrap replicates were used. Data analyses were conducted using R (Version 3.1.0) and SPSS (Version 21). The results are presented for the children in the age range targeted by the product, i.e., between 6 and 23 months, except where otherwise mentioned.

## Results

### Characteristics of the survey sample

A total of 1,113 caregiver-child pairs were surveyed, which corresponds to a response rate of 95.1%. The main characteristics of the survey households and population are presented in Table 1. Among households included in the survey, less than one-quarter were classified as poor by the MPI, and a high proportion had electricity, clean cooking fuel, improved flooring, and a safe drinking water source. A relatively small percentage of households had a child who did not attend school or experienced moderate to severe hunger. Nonetheless, adequate toilet sanitation was lacking in almost one-half of households, and only one-half of caregivers had 5 or more years of schooling.

**Table 1:** Survey Population Demographics and Household Characteristics^a^.

Variable	N	Value
**Household level**		
Household size (number of people)	1,113	6.10 (5.81, 6.38)
Household dependency ratio^b^	1,113	0.58 (0.54, 0.63)
Households with MPI score ≥0.33 (%)	1,106	21.0 (16.6, 26.3)
Electricity (%)	1,106	97.0 (82.1, 99.6)
Clean cooking fuel (%)	1,106	77.7 (70.9, 83.3)
Improved flooring (%)	1,106	98.5 (95.7, 99.5)
Safe drinking water source (%)	1,106	99.0 (98.0, 99.5)
Adequate treatment of drinking water (%)	1,106	4.3 (2.4, 7.6)
Safe toilet sanitation (%)	1,106	55.1 (46.6, 63.3)
Households with any household member 5–14 years not currently attending school (%)	1,106	17.8 (15.0, 21.0)
Households experiencing moderate to severe household hunger (%)	1,106	8.7 (6.2, 12.1)
**Caregiver**		
Age of caregiver (years)	1,113	29.0 (28.4, 29.6)
Caregivers with 5 or more years of schooling (%)	1,106	49.5 (44.2, 54.7)
Caregiver with severe acute malnutrition^c^	1,106	4.4% (3.2%, 6.1%)
**Child**		
Age of child (months)	1,113	11.0 (4.0, 16.0)
Below 6 months of age (%)	1,113	29.8 (26.9, 32.8)
6–11 months of age (%)	1,113	21.2 (18.1, 24.7)
12–23 months of age (%)	1,113	49.0 (46.1, 51.9)
Female children (%)	1,113	46.3 (41.5, 51.2)
Child with severe acute malnutrition^d^	1,106	8.4% (6.2%, 11.3%)

^a^ Mean (95% confidence interval) was used as the measure of central tendency for normally distributed variables; percentages (95% confidence interval) were used for proportions. Median (25^th^ and 75^th^ percentiles) was used for non-normally distributed variables.

^b^ Number of household members under 15 years old and over 64 years old divided by number of household members between 15 and 64 years old.

^c^ Severe acute malnutrition defined as MUAC below 230 mm.

^d^ Severe acute malnutrition defined as MUAC below 110 mm for children under 6 months of age and MUAC below 115 mm for children 6 months of age and above.

Results showed very poor IYCF practices in the surveyed children aged 6–23 months (Table 2). Twenty-five percent of the children were fed adequately as measured by the ICFI. Adequate dietary diversity was found to be lower in poor households than in non-poor households (p<0.05). Among children less than 6 months of age, only 27.7% were exclusively breastfed, and the feeding of PKL products and other fortified instant cereals was extremely low: 0.4% of children under 6 months had ever been given a PKL product.

**Table 2:** Infant and Child Feeding Practices for all, poor and non-poor^a^.

Variable	All	Poor^b^	Non-Poor	p-value
ICFI score	4.66 (4.51, 4.81)	4.56 (4.32, 4.80)	4.69 (4.52, 4.85)	0.319
Children with ICFI score of 6 (%)^c^	25.4% (21.2%, 30.1%)	26.3% (18.3%, 36.3%)	25.2% (20.0%, 31.1%)	0.839
Currently breastfed (%)	56.9% (52.5%, 61.2%)	64.4% (51.9%, 75.2%)	54.7% (49.7%, 59.6%)	0.176
Age-appropriate dietary diversity (%)	62.8% (56.9%, 68.3%)	51.9% (39.8%, 63.9%)	65.9% (60.2%, 71.2%)	0.030
Age-appropriate meal frequency (%)	70.8% (64.6%, 76.2%)	71.3% (63.2%, 78.2%)	70.6% (63.3%, 77.0%)	0.887

^a^ Results presented for all children 6–23 months of age (N = 776). Mean (95% confidence interval) was used as the measure of central tendency for normally distributed variables.

^b^ MPI score ≥ 0.33 is considered at risk of acute poverty.

^c^ ICFI score = 6 is equivalent to good practices based on continued breastfeeding, increased dietary diversity, and increased meal frequency based on child’s age range.

### Product coverage

Coverage results are presented in Table 3. Message coverage and contact coverage were lower in poor households than in non-poor households (75.3% and 87.7%, p<0.05, and 27.9% and 40.3%, p<0.05, respectively). However partial and effective coverage were not significantly different between poor and non-poor households.

**Table 3:** Coverage and Consumption of PKL’s Fortified Complementary Instant Cereals among Children 6–23 Months.

Variable	N^a^	Percentage (95% confidence interval)
Message coverage (%)	777	85.0 (81.6, 87.8)
Contact coverage (%)	777	37.8 (33.3, 42.5)
Partial coverage (%)	756	8.8 (6.4, 11.9)
Effective coverage (%)	756	4.6 (2.9, 7.2)

^a^ The percentage coverage was calculated without missing values.

Message coverage and contact coverage were lower in children from poor households (as shown by coverage ratios less than 1; see Table 4). The situation is slightly different with the coverage ratio among children with poor IYCF practices: The message and partial coverage were not significantly different between children with poor and good IYCF practices; however, the contact coverage and effective coverage was higher among children with poor IYCF practices, with relatively better targeting of children with poor IYCF practices.

**Table 4:** Met Need and Coverage Ratios of PKL’s Fortified Complementary Instant Cereals by Coverage Measure and Risk Group ^a^.

Type of coverage	Risk group	Percent met need^b^	Coverage ratio^c^
Message coverage	Poor household^d^	76.0 (65.9, 85.7)	0.88 (0.76, 0.99)
	Poor IYCF^e^	84.5 (79.9, 88.7)	1.02 (0.92, 1.14)
Contact coverage	Poor household	28.5 (20.0, 38.4)	0.70 (0.47, 0.96)
	Poor IYCF	41.3 (36.8, 46.6)	1.35 (0.99, 1.88)
Partial coverage	Poor household	8.5 (3.4, 15.5)	0.84 (0.33, 1.62)
	Poor IYCF	8.8 (5.7, 12.3)	0.67 (0.33, 1.50)
Effective coverage	Poor household	3.4 (0.5, 8.4)	0.71 (0.09, 2.15)
	Poor IYCF	5.1 (2.8, 7.7)	1.83 (0.61, 8.86)

^a^ All values are percent (95% confidence interval), unless otherwise indicated. Results presented for all children 6–23 months of age.

^b^ Met need is the estimated coverage in the at-risk group. Estimated using blocked weighted bootstrap estimation technique.

^c^ Coverage ratio is the ratio of coverage estimates in at-risk vs. not at-risk groups. Estimated using blocked weighted bootstrap estimation technique.

^d^ MPI score ≥ 0.33 is considered at risk of acute poverty.

^e^ ICFI score < 6 is considered poor IYCF.

The survey also assessed the contact coverage of other products. Fortified instant cereals of other brands were also commonly consumed (69.7%). The most commonly consumed brands were: Cerelac (33.3%), Blédina (30.6%), France Lait (11.0%) and Phosphatine (8.5%). Although the data is cross-sectional, the results suggest that no single product would cover all children. The program implications of this need to be assessed further.

### Program enablers and barriers

The main program enablers were: the widespread utilization and acceptability of products from the fortified complementary instant cereal category, the high awareness of PKL complementary food products, and the perception of fortified complementary food as being healthy for the child. Among children 6–23 months of age included in the survey, 69.6% were commonly fed with any fortified complementary instant cereals. The main sources of information on Farinor and Nutribon were shopkeepers or pharmacists (33.1%), television (22.2%), health workers (13.7%), and acquaintances (13.5%). Among the caregivers interviewed, 31.0% reported that they liked PKL’s products because the product is good for the child, 20.6% because of the taste, 8.8% because of the packaging, and 6.6% because of the price.

The main barriers were: intra-household sharing of the product was common and IYCF practices were poor. For poor households more specifically, the main barrier was the price of the product (45.8% of the poor caregivers reported that the product was too expensive). The practice of intra-household sharing of the product, which reduces the amount available for children in the target age range, was reported in 24.3% of the households. The product was shared mainly with other children in the household (71.9%), adolescents in the households (23.3%), and the caregiver (21.0%).

## Discussion

The findings of this survey show that, despite high awareness of Farinor and Nutribon, regular utilization is low. Poverty was a barrier to message and contact coverage: Respondents in poor households mentioned the high price of the product as a reason for not trying the product. However, the frequency that the product was used did not differ between poor and non-poor groups, indicating that frequent utilization is not dependent on poverty status.

The coverage-need analysis shows inadequate targeting of poor segments of the population suggesting there are high unmet needs in this population group. The contact coverage data of other complementary food products consumed suggest, not surprisingly, that no single complementary food product would cover all children. This finding highlights the importance of further increasing the availability and affordability of fortified complementary foods beyond just a single producer. Working on the national standards for formulation of these products would affect many children living in Abidjan and possibly elsewhere, as the consumption of any brand of fortified complementary instant cereals is relatively common. Furthermore, efforts to this end may include free or subsidized distribution by the public system or by nongovernmental organizations.

This survey found that IYCF practices in the population were poor, which is consistent with findings from other surveys conducted in Côte d’Ivoire [2, 10]. This further emphasizes the major efforts needed to improve IYCF practices, especially with regard to exclusive breastfeeding for children under 6 months and feeding nutrient-rich complementary foods to children over 6 months. To expect the program to have a measurable impact on children’s nutritional status, general IYCF practices would need to be improved, effective coverage would need to be increased, and possibly other interventions (such as improved water, sanitation, and hygiene, and malaria prevention and treatment) would be needed to address factors contributing to various forms of malnutrition [23].

A limitation of this survey is that the results can be generalized only to children residing in Abidjan, not the rest of the country. However, this was the main target of the distribution of Farinor and Nutribon. If and when distribution is expanded to other parts of Côte d'Ivoire, additional evaluation studies should be done to measure acceptability and coverage. In addition, because Farinor and Nutribon are targeted to children under 2 years of age, this survey included only these children in the evaluation. It did not include older children who may also consume fortified instant cereals. Nonetheless, older children are generally at lower risk of malnutrition than younger children.

This survey was focused on program coverage and barriers to coverage. The efficacy of the product has been assessed in a separate cluster-randomized controlled trial [23]. Future work should investigate how a behavior change strategy could be designed to better promote the effective coverage of fortified complementary foods and how to sustainably improve IYCF practices. Additional delivery models should be explored to complement the market-based approach and to increase the coverage and more efficiently target the poorest population groups.

## Conclusion

The results from the present study indicate that availability on the market and high awareness among the target population is not sufficient to achieve high and effective coverage. With market-based delivery models, significant efforts are needed to improve demand. Moreover, given the high prevalence of malnutrition and poor IYCF practices, additional modes of delivering IYCF interventions and improving IYCF practices should be considered.

## Supporting Information

S1 File STROBE Checklist. (DOCX)

S2 File Questionnaire. (PDF)

## References

[1] Rohner F, Northrop-Clewes C, Tschannen AB, Bosso PE, Kouassi-Gohou V, Erhardt JG, et al Prevalence and public health relevance of micronutrient deficiencies and undernutrition in pre-school children and women of reproductive age in Cote d'Ivoire, West Africa. Public Health Nutr. 2014;17(9):2016–28. Epub 2013/11/01. 10.1017/S136898001300222X . 24171836 PMC11107373

[2] Institut National de la Statistique (INS), ICF International. Enquête Démographique et de Santé et à Indicateurs Multiples de Côte d’Ivoire 2011–2012. Calverton, Maryland, USA: INS et ICF International, 2012.

[3] Kramer MS, Kakuma R. Optimal duration of exclusive breastfeeding. Cochrane Database Syst Rev. 2012;8:CD003517 10.1002/14651858.CD003517.pub2 . 22895934 PMC7154583

[4] Wijnhoven TMA, Bollars C, Tabacchi G, Hermoso M. Collate and review data on the composition and volume and intake of breast milk: Results from a systematic literature review. European Micronutrient Recommendations Aligned. 2009.

[5] Meinzen-Derr JK, Guerrero ML, Altaye M, Ortega-Gallegos H, Ruiz-Palacios GM, Morrow AL. Risk of infant anemia is associated with exclusive breast-feeding and maternal anemia in a Mexican cohort. J Nutr. 2006;136(2):452–8. . 16424127 10.1093/jn/136.2.452

[6] Dewey KG, Adu-Afarwuah S. Systematic review of the efficacy and effectiveness of complementary feeding interventions in developing countries. Matern Child Nutr. 2008;4 Suppl 1:24–85. 10.1111/j.1740-8709.2007.00124.x . 18289157 PMC6860813

[7] FAO, WFP, IFAD. The State of Food Insecurity in the World 2012 Economic growth is necessary but not sufficient to accelerate reduction of hunger and malnutrition. Rome: FAO; 2012. 10.3945/an.112.003343 PMC3648734 23319131

[8] Daelmans B, Dewey K, Arimond M, Working Group on I, Young Child Feeding I. New and updated indicators for assessing infant and young child feeding. Food Nutr Bull. 2009;30(2 Suppl):S256–62. . 20496619 10.1177/15648265090302S210

[9] Global Alliance for Improved Nutrition. Case Study: Investing in a Côte d'Ivoire Entrepreneur to Ensure Children's First Foods are Fortified. 2014. Available from: http://www.gainhealth.org/wp-content/uploads/2015/02/Entrepreneur-to-Ensure-Childrens-First-Foods-are-Fortified.pdf (Accessed: 10 October 2016).

[10] Recherche formative sur les pratiques d’alimentation du nourrisson et du jeune enfant (REFACE) PACE/IYCN, Rapport final. Abidjan, Côte d'Ivoire: Helen Keller International-Côte d’Ivoire (HKI-CI), 2010.

[11] 4ème Recensement Général de la Population et de l'Habitat (RGPH) 2014 de Côte d'Ivoire. 2014. Available from: http://www.ins.ci/n/documents/RGPH2014_expo_dg.pdf (Accessed: 10 October 2016).

[12] Kish L. A procedure for objective respondent selection within the household. Journal of the American Statistical Association. 1949;44(247):380–7.

[13] Aaron G, Strutt N, Boateng N, Guevarra E, Siling K, Norris A. Assessing Program Coverage of Two Approaches to Distributing a Complementary Feeding Supplement to Infants and Young Children in Ghana PLoS One. 2016;e 162462 10.1371/journal.pone.0162462 27755554 PMC5068796

[14] Kennedy G, Ballard T, Dop M. Guidelines for measuring household and individual dietary diversity. In: FAO, editor. 2010.

[15] Alkire S, Santos ME. Measuring acute poverty in the developing world: Robustness and scope of the multidimensional poverty index. World Development. 2014;59:251–74.

[16] UNHCR. Guidelines for selective feeding: the management of malnutrition in emergencies. In: UNHCR, editor. 2011.

[17] Ballard T, Coates J, Swindale A, Deitcher M. Household Hunger Scale: Indicator Definition and Measurement Guide2011.

[18] Guevarra E, Siling K, Chiwile F, Mutunga M, Senesie J, Beckley W, et al IYCF assessment with small-sample surveys—A proposal for a simplified and structured approach (www.ennonline.net/fex/47/iycf). Field Exchange. 2014;47.

[19] Tanahashi T. Health service coverage and its evaluation. Bull World Health Organ. 1978;56(2):295–303. 96953 PMC2395571

[20] Aaron GJ, Sodani PR, Sankar R, Fairhurst J, Siling K, Guevarra E, et al Household Coverage of Fortified Staple Food Commodities in Rajasthan, India. PLoS One. 2016;11(10):e0163176 10.1371/journal.pone.0163176 27760123 PMC5070859

[21] Lauritsen JM, Bruus M. EpiData Entry v3.1: A comprehensive tool for validated entry and documentation of data. Association TE, editor: Odense Denmark; 2004.

[22] Cameron A, Gelbach J, Miller DD. Bootstrap-based improvements for inference with clustered errors. Rev Econ Stat. 2008;(90):414–27.

[23] Glinz D, Hurrell RF, Ouattara M, Zimmermann MB, Brittenham GM, Adiossan LG, et al The effect of iron-fortified complementary food and intermittent preventive treatment of malaria on anaemia in 12- to 36-month-old children: a cluster-randomised controlled trial. Malar J. 2015;14(1):347 10.1186/s12936-015-0872-3 26377199 PMC4573684

