# Supplementary material for: High Awareness but Low Coverage of a Locally Produced Fortified Complementary Food in Abidjan, Côte d’Ivoire: Findings from a Cross-Sectional Survey
Source: PLoS One. 2016 Nov 8;11(11):e0166295. doi: 10.1371/journal.pone.0166295 (PMC5100976; doi:10.1371/journal.pone.0166295)
Supplement: S2 File — (PDF) [file pone.0166295.s002.pdf]

ABIDJAN COVERAGE SURVEY 2014

HOUSEHOLD QUESTIONNAIRE

dateint	Date of interview	DD / MM / YY	__ __ / __ __ / 1 4		
teamid	Team identifier	__ __	intid	Interviewer identifier	__ __
commid	Commune	1. Abobo<br>2. Adjamé<br>3. Attécoubé<br>4. Cocody<br>5. Koumassi	6. Marcory<br>7. Plateau<br>8. Port-Bouet<br>9. Treichville<br>10. Yopougon		__ __
neiname	Neighborhood name	________________________________			
psu	Cluster identifier				__ __ __ __
hh	Household identifier				__ __ __

Good morning / Good evening Madam / Sir,

My name is [NAME OF INTERVIEWER] and I work for the CSRS (Centre Suisse de Recherches Scientifiques en Côte d'Ivoire). We are currently conducting a survey in Abidjan on the use of different food products in the household and in particular among children under 2 years of age. We also want to gather some information on the coverage of fortified complementary foods developed through a partnership of Helen Keller International and Protein Kissèe-La.

The first part of the interview will be focusing on the composition of the household, including all its members. Then, based on this information, I would like to interview the mother or caregiver of the child of less than 2 years of age. If there is more than one child of less than 2 years of age, I will select one at random. I will then ask the woman/caregiver of the child what she and the child have eaten yesterday, and some feeding practices. I will measure the mid-upper arm circumference of the child and the mother/caregiver to assess their nutritional status. At the end, I will ask for some oil and salt samples if available.

The questions to you will take about 15 minutes and the questions on the child will take about 40 minutes.

Do you agree to start with the first part of the interview?

cons1	Oral consent to fill in the household roster obtained ?	Yes / No	If **yes**, start<br>If **no**, stop here

HOUSEHOLD ROSTER						
Household line (hhl)	Name	Sex (sex)	Age *(years OR months)*		ONLY for persons aged > 5 years	
			Years (agey)	Months (agem)	Currently attending school or college? (sch)	5 or more years of education? (edu)
1		M / F			Yes / No	Yes / No
2		M / F			Yes / No	Yes / No
3		M / F			Yes / No	Yes / No
4		M / F			Yes / No	Yes / No
5		M / F			Yes / No	Yes / No
6		M / F			Yes / No	Yes / No
7		M / F			Yes / No	Yes / No
8		M / F			Yes / No	Yes / No
9		M / F			Yes / No	Yes / No
10		M / F			Yes / No	Yes / No
11		M / F			Yes / No	Yes / No
12		M / F			Yes / No	Yes / No
13		M / F			Yes / No	Yes / No
14		M / F			Yes / No	Yes / No
15		M / F			Yes / No	Yes / No

und2	Total number of children under 2 years old in the household	

Sélection aléatoire de l’enfant et sa mère → Tableau de Kish

Age of child (months)	Name of child	Number of household line for child	Number of household line for caregiver

**Line child (rand1)**	**Line caregiver (rand2)**

Written consent of the caregiver obtained?

**cons 2**	Yes / No	If **yes**, start<br>If **no**, stop here

HOUSEHOLD CHARACTERISTICS (1)			
N°	QUESTIONS	ANSWERS	SKIPS
mpi1	Does your household have electricity?	Yes / No	
mpi2	What fuel does your household <u>mainly</u> use for cooking?<br><br>*Circle only **<u>one</u>** choice.*	Electricity...1<br>LPG...2<br>Natural gas...3<br>Biogas..4<br>Kersosene...5<br>Coal / Lignite...6<br>Charcoal..7<br>Wood...8<br>Straw / Shrubs / Grass......................................9<br>Agricultural crop..10<br>Animal dung...11<br>No food cooked in household.........................12<br>Don't know...88<br>Other: ______________________ 99	
mpi3	What is the main material of the floor of the dwelling?<br><br>***Observation.***<br><br>*Circle only **<u>one</u>** choice.*	Natural floor<br>Earth / sand..1<br>Dung...2<br>Rudimentary floor<br>Wood planks...3<br>Palm / bamboo...4<br>Finished floor<br>Parquet / polished wood..............................5<br>Vinyl / asphalt strips....................................6<br>Ceramic tiles...7<br>Cement...8<br>Carpet...9<br>Other: ______________________ 99	
dhs1	What is the main material of the roof of the dwelling?<br><br>***Observation.***<br><br>*Circle only **<u>one</u>** choice.*	Natural roofing<br>No roofing..1<br>Thatch / palm leaves....................................2<br>Sod...3<br>Rudimentary roofing<br>Rustic mat...4<br>Palm / bamboo...5<br>Wood planks...6<br>Cardboard...7<br>Finished roofing<br>Metal...8<br>Wood...9<br>Calamine / cement fiber..............................10<br>Ceramic tiles..11<br>Cement...12<br>Roofing shingles..13<br>Other: ______________________ 99	

HOUSEHOLD CHARACTERISTICS (2)			
N°	QUESTIONS	ANSWERS	SKIPS
dhs2	What is the main material of the exterior walls of the dwelling?<br><br>***Observation.***<br><br>*Circle only **one** choice.*	Natural walls<br>No walls..1<br>Cane / palm / trunks...2<br>Dirt..3<br>Rudimentary walls<br>Bamboo with mud...4<br>Stone with mud..5<br>Uncovered adobe...6<br>Plywood...7<br>Cardboard..8<br>Reused wood...9<br>Finished walls<br>Cement...10<br>Stone with lime / cement....................................11<br>Bricks...12<br>Cement blocks...13<br>Covered adobe..14<br>Wood planks / shingles......................................15<br>Other: ______________________ 99	
dhs3	Do you or anyone in this household own this dwelling ?	Yes / No	
dhs4	In this household, how many rooms do you use for sleeping?	Number of rooms ☐ ☐	
mpi4	Does your household or anyone in the household have ... ?<br><br>***Prompt for each item**; record all items owned by household or a member...*<br><br>*Circle Yes or No for **each** item.*	A. Radio — Yes / No	
		B. Television — Yes / No	
		C. Mobile telephone — Yes / No	
		D. Non-mobile telephone — Yes / No	
		E. TV5 antenna — Yes / No	
		F. Cable subscription — Yes / No	
		G. Video / CD / DVD player — Yes / No	
		H. Internet — Yes / No	
		I. Computer — Yes / No	
		J. Watch or clock — Yes / No	
		K. Bicycle or tricycle — Yes / No	
		L. Motorcycle or scooter — Yes / No	
		M. Car, truck, or jeep — Yes / No	
		N. Refrigerator — Yes / No	
		O. Dish washer / washing machine — Yes / No	
		P. Cooking stove (electric or gaz) — Yes / No	
		Q. Improved cooking space — Yes / No	
		R. Air conditioner — Yes / No	
		S. Canoe — Yes / No	
		T. Boat with motor — Yes / No	
		U. Cart — Yes / No	
		V. Animal-drawn cart — Yes / No	
		W. Bank or post-office account — Yes / No	

WASH, SANITATION, AND HYGIENE (WASH) (1)			
N°	QUESTIONS	ANSWERS	SKIPS
w1	What is the <u>main</u> source of drinking water for the members of your household?<br><br>*Circle only **one** choice.*	Piped water<br>Piped into dwelling....................................,.........1<br>Piped to yard / plot..2<br>Public tap / standpipe...3<br>Tube well or borehole..4<br>Dug well<br>Protected dug well..5<br>Unprotected dug well...6<br>Water from spring<br>Protected spring...7<br><u>Un</u>protected spring...8<br>Rainwater..9<br>Tankertruck..10<br>Cart with small tank...11<br>Surface water (river / dam / lake / pond / stream / canal / irrigation channel)..12<br>Bottled / sachet water..13<br>Don't know...88<br>Other: ______________________ 99	
w2a	Do you <u>usually</u> do anything to your drinking water to make it safer to drink?	Yes / No	If **No**, skip to **w3**
w2b	What do you <u>usually</u> do to the water to make it safer to drink?<br><br>*Do not prompt. Probe "Anything else?".*<br><br>*Circle Yes for each item mentioned and No for each item not mentioned.*	A. Boil — Yes / No<br>B. Add bleach / chlorine tablet — Yes / No<br>C. Strain through a cloth — Yes / No<br>D. Use a water filter (ceramic / sand / composite/...) — Yes / No<br>E. Solar disinfection — Yes / No<br>F. Let it stand and settle — Yes / No<br>G. Don't know — Yes / No<br>H. Other: ______________________ — Yes / No	
w3	What kind of toilet facility do members of your household usually use?<br><br>*Do **not** prompt.*<br><br>*Circle only **one** answer.*	Flush or pour flush toilet<br>Flush or pour flush to piped sewer system.............1<br>Flush or pour flush to septic tank.............................2<br>Flush or pour flush to pit latrine...............................3<br>Flush or pour flush to elsewhere..............................4<br>Flush or pour flush, don't know where.....................5<br>Pit latrine<br>Ventilated improved pit latrine................................6<br>Pit latrine <u>with</u> slab...7<br>Pit latrine <u>without</u> slab / open pit............................8<br>Composting toilet..9<br>Bucket toilet..10<br>Hanging toilet / hanging latrine...............................11<br>No facilities / bush / field...12<br>Don't know...88<br>Other: ______________________ 99	
w4	Do you share this facility with other households?	Yes / No	

WASH, SANITATION, AND HYGIENE (WASH) (2)			
N°	QUESTIONS	ANSWERS	SKIPS
w5	The <u>last time</u> [NAME OF CHILD] passed stools, what was done to dispose the stools?<br><br>*Do **not** prompt.*<br><br>*Circle only **one** answer.*	Child used toilet / latrine...1<br>Put into toilet / latrine..2<br>Buried..3<br>Put into drain or ditech...4<br>Thrown into garbage...5<br>Nothing / left in open..6<br>Don't know / Don't remember ....8<br>Other: ________________________ 9	
w6	Please tell me all of the occasions when it is important for you to wash hands...<br><br>*Do **not** prompt. Probe "Anything else?".*<br><br>*Circle Yes for each item mentioned and No for each item not mentioned.*	A. Before eating Yes / No<br>B. Before feeding a child Yes / No<br>C. Before cooking / preparing food Yes / No<br>D. After defecation / urination Yes / No<br>E. After cleaning a child that has defecated / changing nappes / washing diaper Yes / No<br>F. Don't know Yes / No<br>G. Other : ________________________ Yes / No	

HOUSEHOLD HUNGER SCALE (FOOD SECURITY)			
N°	QUESTIONS	ANSWERS	SKIPS
hh1	How many times in the last month was there ever no food to eat of any kind in your house because of lack of resources to get food?<br><br>*If 'none', record 00.*	Number of times ☐☐	
hh2	How many times in the last month did you go to sleep at night hungry because there was not enough food?<br><br>*If 'none', record 00.*	Number of times ☐☐	
hh3	How many times in the last month did you go a whole day and night without eating anything at all because there was not enough food?<br><br>*If 'none', record 00.*	Number of times ☐☐	

SHORT BIRTH HISTORY (MPI MORTALITY)			
N°	QUESTIONS	ANSWERS	SKIPS
The purpose of the short birth history (SBH) component is to determine whether any child that was recently born (<u>during the last 3 births, during that last 5 years</u>) to the respondent has died.			
d1	*Tell the caregiver (mother) :*<br>- I will be asking about your recent births.<br>- I am interested in the three most recent births.<br>- I am interested in all children that were <u>born</u> <u>alive</u>.<br><br>*Ask the following questions :*<br>- When was your most recent birth?<br>- Where is this child now?<br>- Did you have a birth before this most recent one?<br>- Where is this child now?<br>- Did you have a birth before that one?<br>- Where is this child now?	A recently born child has died?<br><br>Yes / No / N/A	

INFANT AND YOUNG CHILD FEEDING (IYCF)			
N°	QUESTIONS	ANSWERS	SKIPS
f1	Is [NAME OF CHILD] currently breastfed?	Yes / No	
f2	Does [NAME OF CHILD] take any food or drink other than breastmilk?	Yes / No	
f3	How many times was [NAME OF CHILD] fed mashed or pureed food or solid or semi-solid food as a meal or a snack since this time yesterday?	Number of times ☐☐	

MOTHER AND CHILD DIETARY DIVERSITY			
**<u>Since this time yesterday</u>**, what food did you and [NAME OF CHILD] eat?<br><br>*Probe for items not mentioned:* Did you and [NAME OF CHILD] have any of the following things to eat or drink?<br><br>*Circle **Yes** if eaten since this time yesterday, and **No** if not eaten.*			
N°	ITEMS	A. Caregiver	B. Child
dd01	Plain water	Yes / No	Yes / No
dd02	Tinned or powdered milk<br>Tinned or powdered infant formula (Bledilait, Nido, Nan, Lactogen, Eveil, Ninolac, Lailac, Célia, Dano) or any other milk (excluding breast milk)	Yes / No	Yes / No
dd03	Sweetened or flavoured water, minerals, malt drinks, tea, coffee, liquor, beer, soda	Yes / No	Yes / No
dd04	Any food made from grain (maize, millet, wheat, fonio, sorghum, rice)	Yes / No	Yes / No
dd05	Any food made from fruits or vegetables that have yellow or orange flesh (carrots, mangoes, papaya, squash, melon, and 100% fruit juices made from these fruits)	Yes / No	Yes / No
dd06	Any dark green leafy vegetables (spinach, cassava leaves, amaranth, potato leaves, moringa leaves, okra leaves)	Yes / No	Yes / No
dd07	Any food made from roots or tubers (potatoes, sweet potatoes, yams, cassava, malanga)	Yes / No	Yes / No
dd08	Any food made from beans, peas, nuts, or seeds (palm seed, peanuts, soybeans, sesame)	Yes / No	Yes / No
dd09	Any other fruits or vegetables (coconut, eggplant, tomatoes, peppers, zucchini, avocado, lemon, banana, plantain, orange, mandarin, apple, cucumber, okra, onion) and 100% fruit juices (orange, passion, roselle, lemon, ginger, baobab)	Yes / No	Yes / No
dd10	Liver, kidney, heart, or other organ meats	Yes / No	Yes / No
dd11	Any meat (beef, pork, lamb, mutton, goat, chicken, duck, guinea fowl)	Yes / No	Yes / No
dd12	Fresh or dried fish, shellfish, or seafood, snails, crabs (makerel, sea bream, capitaine, carp, shrimp fish, shrimps)	Yes / No	Yes / No
dd13	Cheese, yoghurt, or other milk products (Kiri, La Vache Qui Rit, Yoplait, Vigor, Petit Suisse, Elle & Vie)	Yes / No	Yes / No
dd14	Eggs (from chicken, quail)	Yes / No	Yes / No
dd15	Sugary foods (sugar cane, sweets, candies, chocolate, cakes, biscuits)	Yes / No	Yes / No
dd16	Any food made with oil, fat, or butter	Yes / No	Yes / No
dd17	Red palm oil	Yes / No	Yes / No

FORTIFIED COMPLEMENTARY FOODS (1)			
N°	QUESTIONS	ANSWERS	SKIPS
cf1	Have you ever seen or heard of Farinor or Nutribon?<br><br>*Show the pictures of Farinor and Nutribon.*	Yes / No	If **No**, skip to **cf16**
cf2	Where did you hear of Farinor / Nutribon from?<br><br>*Do **not** prompt. Probe "Anything else?".*<br><br>*Circle Yes for each item mentioned and No for each item not mentioned.*	A. TV Yes / No	
		B. Radio Yes / No	
		C. Billboards / wallpapers / painted walls Yes / No	
		D. Mobile van activation Yes / No	
		E. Church meeting Yes / No	
		F. Crèche / nursery Yes / No	
		G. Health clinic / health worker / IYCF mobiliser Yes / No	
		H. Community leaders Yes / No	
		I. Relative / friend / neighbour Yes / No	
		J. Shopkeeper / pharmacy Yes / No	
		K. Social media Yes / No	
		L. Market place / Market Yes / No	
		M. Don't know / Don't remember Yes / No	
		N. Other: ___________________ Yes / No	
cf3	Have you ever given Farinor/Nutribon to [NAME OF CHILD] before?	Yes / No	If **Yes**, skip to **cf5**
cf4	Why have you never used Farinor/Nutribon before?<br><br>*Do **<u>not</u>** prompt. Probe "Anything else?".*<br><br>*Circle Yes for each item mentioned and No for each item not mentioned.*	A. Too expensive Yes / No	Skip to **cf16**
		B. Not available in the market Yes / No	
		C. Do not see a need for the product Yes / No	
		D. Heard of bad experience of someone else Yes / No	
		E. Advised not to use it Yes / No	
		F. Not seen other mothers use it Yes / No	
		G. Don't trust the product Yes / No	
		H. Haven't seen it Yes / No	
		I. Child too young Yes / No	
		J. Other family members discourage using it Yes / No	
		K. Don't know / Don't remember Yes / No	
		L. Other: _______________________ Yes / No	
cf5	When was the <u>last time</u> you fed [NAME OF CHILD] with Farinor/Nutribon?<br><br>*Start with '1' if days and '2' if months.*<br>*If 'don't know', write in '888'.*	... days or months ago ☐ ☐☐	

FORTIFIED COMPLEMENTARY FOODS (2)			
N°	QUESTIONS	ANSWERS	SKIPS
cf6	This <u>last time</u> you fed [NAME OF CHILD] with Farinor/Nutribon, how much did you feed him?<br><br>*Start with '1' if soup spoon, '2' if tablespoon, and '3' if 50g sachet.*<br>*If 'don't know', record '888'.*	Quantity ☐ ☐ ☐	
cf7	This <u>last time</u> you fed [NAME OF CHILD] with Farinor/Nutribon, at which mealtime(s) did you give it?<br><br>*Circle Yes for each item mentioned and No for each item not mentioned.*	A. Breakfast Yes / No<br>B. Morning snack Yes / No<br>C. Lunch Yes / No<br>D. Afternoon snack Yes / No<br>E. Dinner Yes / No<br>F. Evening snack Yes / No<br>G. Don't know / Don't remember Yes / No<br>H. Other: __________________ Yes / No	
cf8	This <u>last time</u> you fed [NAME OF CHILD] with Farinor/Nutribon, did you add anything to it, apart from water?	Yes / No	If **no**, skip to **cf10**
cf9	What did you add to Farinor/Nutribon, apart from water?<br><br>*Do **not** prompt. Probe "Anything else?"*<br><br>*Circle Yes for each item mentioned and No for each item not mentioned.*	A. Salt Yes / No<br>B. Sugar Yes / No<br>C. Milk Yes / No<br>D. Honey or jam Yes / No<br>E. Oil Yes / No<br>F. Don't know / Don't remember Yes / No<br>G. Other: __________________ Yes / No	
cf10a	Does anybody else in the household currently eat Farinor/Nutribon?	Oui / Non	If **no**, skip to **cf11**
cf10b	If yes, who else?<br><br>*Do **not** prompt. Probe "Anybody else?"*<br><br>*Circle Yes for each item mentioned and No for each item not mentioned.*	A. Other children in the household Yes / No<br>B. Adolescents in the household Yes / No<br>C. Me (mother / principal caregiver) Yes / No<br>D. Other adults in the household Yes / No<br>E. Don't know / Don't remember Yes / No<br>F. Other: ___________________ Yes / No	
cf11	The <u>last time</u> you got Farinor/Nutribon, where did you get it from?<br><br>*Circle only **one** answer.*	Supermarket..1<br>Shop / superette..2<br>Boutique / Kiosk...3<br>Market / Street stand.....................................4<br>Moving street vendor.....................................5<br>Pharmacy...6<br>Neighbour / relatives / friends.........................7<br>Health worker...8<br>NGO / religious association............................9<br>Don't know / Don't remember.......................88<br>Other: _______________________............99	

FORTIFIED COMPLEMENTARY FOODS (3)			
N°	QUESTIONS	ANSWERS	SKIPS
cf12	The <u>last time</u> you got Farinor/Nutribon, how much did you get?<br><br>*Start with '1' if 400g box, '2' if 250g box, and '3' if 200g box or sachet, and '4' if 50g sachet.*<br>*If 'don't know', record '888'.*	Quantity ☐☐☐	
cf13	The <u>last time</u> you got Farinor/Nutribon, how much did you pay?<br><br>*If 'gift', record 00000.*<br>*If 'don't know', record 88888.*	Francs CFA ☐☐☐☐☐	
cf14	What do you like about Farinor/Nutribon?<br><br>*Do **not** prompt. Probe "Anything else?"*<br><br>*Circle Yes for each item mentioned and No for each item not mentioned.*	A. Taste Yes / No<br>B. Packaging Yes / No<br>C. Price Yes / No<br>D. How it is promoted / advertised Yes / No<br>E. Good for the health of children Yes / No<br>F. Easy availability Yes / No<br>G. Trustworthy source Yes / No<br>H. Locally produced Yes / No<br>I. Nothing Yes / No<br>J. Don't know / Don't remember Yes / No<br>K. Other: ____________________ Yes / No	
cf15	What do you <u>not</u> like about Farinor/Nutribon?<br><br>*Do **not** prompt. Probe "Anything else?"*<br><br>*Circle Yes for each item mentioned and No for each item not mentioned.*	A. Taste Yes / No<br>B. Packaging Yes / No<br>C. Price Yes / No<br>D. How it is promoted / advertised Yes / No<br>E. Not good for the health of children Yes / No<br>F. Not easy availability Yes / No<br>G. Untrustworthy source Yes / No<br>H. Locally produced Yes / No<br>I. Nothing Yes / No<br>J. Don't know / Don't remember Yes / No<br>K. Other: ____________________ Yes / No	

FORTIFIED COMPLEMENTARY FOODS (4)			
N°	QUESTIONS	ANSWERS	SKIPS
cf16	Do you currently give any of these other products to [NAME OF CHILD]?<br><br>*Probe for items not mentioned.*<br><br>*Circle Yes for each item mentioned and No for each item not mentioned.*	A. Cerelac Yes / No	
		B. Nutrilac Yes / No	
		C. France Lait Yes / No	
		D. Blédine / Blédina Yes / No	
		E. Ninolac Yes / No	
		F. Phosphatine Yes / No	
		G. Picot Yes / No	
		H. Lailac Yes / No	
		I. Celia Yes / No	
		J. Nutrilon Yes / No	
		K. Babylac Yes / No	
		L. Home-made gruel Yes / No	
		M. Mashed family foods Yes / No	

MOTHER AND CHILD HEALTH AND NUTRITION DATA			
N°	QUESTIONS	ANSWERS	REMARKS
MOTHER			
fefol	Have you taken iron tablets during your pregnancy with [NAME OF CHILD]?<br><br>*Circle 'N/A', if the caregiver is not the mother of the child.*	Yes / No / N/A	
muacm	*Take the MUAC of the **mother** on her left arm*<br><br>*If 'refused,' write' 666'.*<br>*If arm is too big, write '777'.*<br>*If the respondent is a man, write '555'.*	mm ☐☐☐	*If MUAC **< 185mm*** ***→ Refer!***
CHILD			
vas	Has [NAME OF CHILD] taken a vitamin A capsule in the past 6 months?<br><br>*Show the vitamin A capsule.*	Yes / No / Don't know	
muacc	*Take the MUAC of the **child** on his/her left arm*<br><br>*If 'refused' write 666.*	mm ☐☐☐	*If*<br>*<6 months and*<br>*MUAC **< 110 mm***<br>*OR*<br>*>6 months and*<br>*MUAC **< 115 mm***<br>***→ Refer!***
bcg	*Check for BCG scar on left arm*	Yes / No	
oed	*Check for oedema*	Yes / No	*If **oedema present*** ***→ Refer!***
hc	*Check for child health booklet*	Yes / No	

After these questions concerning you and [NAME OF CHILD], I would now like to ask you a few questions that concern the WHOLE household again.

OIL FORTIFICATION COVERAGE (1)			
N°	QUESTIONS	ANSWERS	SKIPS
of1	What is the main edible oil consumed by your household (*clarify with: the oil that you use on most days in most meals in the home*)?<br><br>*Circle only **one** answer.*	Refined palm oil..1<br>Soybean oil...2<br>Arachnid oil..3<br>Sunflower oil...4<br>Olive oil...5<br>Vegetable blend oil...................................6<br>Don't know / Don't remember...................8<br>Other: ________________________......9	
of2	Can you tell me where you usually get this [MAIN OIL TYPE]?<br><br>*Circle only **one** answer.*	Purchased from supermarket.................1<br>Purchased from shop / superette............2<br>Purchased from boutique / kiosk.............3<br>Purchased from market / street stand.....4<br>Purchased from moving street vendor ...5<br>Made it at home.......................................6<br>Received from food aid............................7<br>Don't know / Don't remember...................8<br>Other: ________________________......9	If '**Made it at home**' (6), skip to next page
of3	This [MAIN OIL TYPE] that you consume, when you get it, is it usually packaged or open?<br><br>*Open means packaged in another bottle than the original one, in a sachet or from a big container.*	Packaged...1<br>Open...2<br>Don't know / Don't remember..................8<br>Other: ________________________......9	
of4	Can you tell me the brand of this oil?<br><br>*Circle only **one** answer.*	Dinor...1<br>Palme d'Or..2<br>Goutte d'Or...3<br>Huilor..4<br>Casino...5<br>Turna...6<br>Borgesol..7<br>Fleuriol..8<br>Aromate...9<br>Lesieur..10<br>Viking...11<br>Goudor...12<br>Don't know / Don't remember................88<br>Other: ________________________....99	

OIL FORTIFICATION COVERAGE (2)			
N°	QUESTIONS	ANSWERS	SKIPS
of5	The **last time** your household [MAIN OIL TYPE], how much did you get?<br><br>*Start with ‘1’ if liters, ‘2’ if mililiters, and ‘3’ if FCFA.*<br><br>*If ‘don’t know’, record ‘8888’.*	Quantity ☐☐☐☐	
of6	How long does this amount usually last in your household?<br><br>*Start with ‘1’ if days and ‘2’ if months.*<br><br>*If ‘don’t know’, record ‘888’.*	Duration ☐☐☐	
of7	May I take a small sample?<br><br>*If **yes**, stick label, matching to sample, here.*<br><br>*If **no**, please write the reason (e.g. no oil at the household at the present moment)*	*STICK LABEL HERE*	

FLOUR FORTIFICATION COVERAGE			
N°	QUESTIONS	ANSWERS	SKIPS
ff1	What is the <u>main</u> edible <u>flour</u> consumed by your household (*clarify with: the oil that you use on most days in most meals in the home*)?<br><br>*Circle only **one** answer.*	Wheat flour...1<br>Maize flour..2<br>Millet flour...3<br>Sorghum flour...4<br>Fonio flour...5<br>Don't use...7<br>Don't know / Don't remember..................8<br>Other: ________________________.......9	If '**Don't use'** (7), skip to **fc01**.
ff2	Can you tell me where you usually get this [MAIN FLOUR TYPE]?<br><br>*Circle only **one** answer.*	Purchased from supermarket.................1<br>Purchased from shop / superette............2<br>Purchased from boutique / kiosk.............3<br>Purchased from market / street stand.....4<br>Purchased from moving street vendor ...5<br>Made it at home......................................6<br>Received from food aid............................7<br>Don't know / Don't remember..................8<br>Other: ________________________......9	If '**Made it at home**' (6), skip to **fc01**
ff3	Can you tell me the brand of this flour?<br><br>*Circle only **one** answer.*	GMA...1<br>LMCI...2<br>MMCI..3<br>Casino...4<br>Doucine...5<br>Don't know / Don't remember..................8<br>Other: ________________________.......9	
ff4	The <u>last time</u> your household [MAIN FLOUR TYPE], how much did you get?<br><br>*Start with '1' if kg, '2' if g, and '3' if CFA.*<br><br>*If 'don't know', record '8888'.*	Quantity ☐☐☐☐	
ff5	How long does this amount usually last in your household?<br><br>*Start with '1' if days and '2' if months.*<br><br>*If 'don't know', record '888'.*	Duration ☐☐☐	

INDIVIDUAL FLOUR CONSUMPTION (1)						
**In the last 7 days**, how many times did you and [NAME OF CHILD] eat products made from wheat flour, such as [food item]?<br><br>*If frequency = **00**, don't ask the portion size!*<br><br>Usually how much of [food item] did you and [NAME OF CHILD] eat at one sitting? *Show pictures of portions!*						
N°	ITEMS	A. Caregiver		B. Child		
		1. Frequency (# times)	2. Portion size	1. Frequency (# times)	2. Portion size	
fc01	Baguette					
fc02	Ball-bread					
fc03	Campagne bread					
fc04	Milk bread					
fc05	Raisin bread					
fc06	Croissant					
fc07	Chocolate bread					
fc08	Sweet bread 500F					
fc09	Sweet bread 200F					
fc10	Sweet bread 100F					
fc11	Sweet bread 50F					
fc12	Sweet white bread 50F					
fc13	Sweet rond bread					
fc14	Cake 50F					
fc15	Cake 100F					
fc16	Galettes aller-retour					
fc17	Galettes white					
fc18	Galettes yellow 10 F					
fc19	Galettes yellow 25 F					
fc20	Galettes (Gbofroto)					
fc21	Gbozon					
fc22	Others: ____________________		cfa			

SALT FORTIFICATION COVERAGE			
N°	QUESTIONS	ANSWERS	SKIPS
sf1	The <u>last time</u> your household purchased <u>**salt**</u>, how was it packaged?	Packaged salt..1<br>Open salt..2<br>Don't know / Don't remember.................8	
sf2	Can you tell me the brand?<br><br>*Specify*	Salina..1<br>SAGID...2<br>Baleine..3<br>Sea salt...4<br>Don't know / Don't remember..................8<br>Other:_______________.........................9	
sf3	May I take a small sample?<br><br>*If **yes**, stick label, matching to sample, here.*<br>*If **no**, write in the reason (e.g. no salt available in the household)*	*STICK LABEL HERE*	

BOUILLON CUBE FORTIFICATION COVERAGE			
N°	QUESTIONS	ANSWERS	SKIPS
bcf1	What is the <u>**main**</u> brand of <u>**bouillon cube**</u> consumed by your household (*clarify with: the brand of the bouillon cube that you use on most days in most meals in the home*)?<br><br>*Specify*	Maggi..1<br>Jumbo...2<br>Doli..3<br>Casino...4<br>Adja...5<br>Mamita..6<br>Jocomo...7<br>Maxi gout..8<br>Don't use...77<br>Don't know / Don't remember................88<br>Other:_______________.......................99	If '**don't use**', **end** here
bcf2	The <u>**last time**</u> your household got [BOUILLON CUBE BRAND], how much did you get?<br><br>*If 'don't know', record '88'.*	Number of cubes/sachets ☐☐	
bcf3	How long does this amount usually last in your household?<br><br>*Start with '1' if days and '2' if months.*<br><br>*If 'don't know', record '888'.*	Duration ☐☐☐	

obs	*Please write in any other remarks*		

*** CHECK THE QUESTIONNAIRE & THANK THE MOTHER!***
